# Room-temperature liquid diffused separation induced crystallization for high-quality perovskite single crystals

**DOI:** 10.1038/s41467-020-15037-x

**Published:** 2020-03-04

**Authors:** Fang Yao, Jiali Peng, Ruiming Li, Wenjing Li, Pengbin Gui, Borui Li, Chang Liu, Chen Tao, Qianqian Lin, Guojia Fang

**Affiliations:** Key Lab of Artificial Micro- and Nano-Structures of Ministry of Education of China, School of Physics and Technology, Wuhan University, 430072 Wuhan, PR China

**Keywords:** Chemistry, Materials science, Materials for devices

## Abstract

Large single crystals serve as an ideal platform for investigating intrinsic material properties and optoelectronic applications. Here we develop a method, namely, room-temperature liquid diffused separation induced crystallization that uses silicone oil to separate the solvent from the perovskite precursors, to grow high-quality perovskite single crystals. The growth kinetics of perovskite single crystals using this method is elucidated, and their structural and optoelectronic properties are carefully characterized. The resultant perovskite single crystals, taking CH_3_NH_3_PbBr_3_ as an example, exhibit approximately 1 µs lifetime, a low trap density of 4.4 × 10^9^ cm^−3^, and high yield of 92%, which are appealing for visible light or X-ray detection. We hope our findings will be of great significance for the continued advancement of high-quality perovskite single crystals, through a better understanding of growth mechanisms and their deployment in various optoelectronics. The diffused separation induced crystallization strategy presents a major step forward for advancing the field on perovskite single crystals.

## Abstract (web summary)

Perovskites are appealing for optoelectronics, but high-quality perovskite single crystals should be grown at low temperature to minimize trap density. Here, the authors report a room-temperature liquid-diffused-induced crystallization for growth of high-quality hybrid perovskite single crystals.

## Introduction

Organic–inorganic trihalide perovskites APbX_3_ (A = CH_3_NH_3_^+^, H_2_NCHNH_2_^+^, Cs^+^ X = Cl^−^, Br^−^, I^−^) have made tremendous progress in optoelectronics, e.g., solar cells^1–5^, light-emitting diodes^6–8^, lasers, and photodetectors (PDs)^9–14^. This is attributed to their excellent optoelectronic properties, e.g., high absorption coefficients over tunable and wide wavelength range, ambipolar charge transport with long and balanced electron–hole diffusion lengths, high carrier mobility, and large dielectric constant^15,16^. Perovskite polycrystalline thin films can be easily deposited with low-temperature solution processes with lost cost regardless of either flat or wrinkled substrates^13,17,18^. Compared with its polycrystalline films, perovskite single crystals (SCs) present none grain boundaries, lower recombination centers, and trap density^19–21^. Moreover, they are more environmentally stable^22^. As a semiconductor crystalline material, SCs are frequently employed as an ideal platform for investigating their optoelectronic properties^23–26^.

Up to now, large hybrid perovskite SCs have been prepared with various solution-based methods, including inverse temperature crystallization (ITC)^27,28^, temperature lowering method^29^, top-seeded solution-growth method^20^, antisolvent vapor-assisted crystallization (AVC)^19^, Bridgman growth method^30^, cavitation-triggered asymmetrical crystallization (CTAC), and low-temperature-gradient crystallization (LTGC)^25,31^. These growth routes for perovskite SCs have made great progress. Optoelectronic applications based on SCs, e.g., solar cells^23,32^, detectors^11,12,25,33,34^, have exhibited superior performances. Bakr and coworkers reported perovskite SC solar cells with power conversion efficiencies reaching up to 21%^32^, Liu et al. fabricated a PD based on perovskite SCs with detectivity as high as 10^13^ Jones^25^. Huang and coworkers reported high radiation and imaging detection devices with sensitivity up to 2.1 × 10^4^ µC Gy_air_^−1^ cm^−2^ under 8 KeV X-ray radiation with perovskite SCs^35,36^. Among these approaches, a rapid facile crystallization route based on the “inverse solubility” effect has been widely used^27,29,37^. It produced perovskite SCs with 100 mm in several hours, one order of magnitude faster than the other methods^19,20,38^. However, the way to obtain supersaturated perovskite solutions in those methods is upon either temperature or antisolvent. The convective currents arising from thermal gradients in growth solution inevitably disturb the ordered growth, leading to undesired twining defects and cracks in the bulk of perovskite SCs^19,39,40^. Moreover, the solubility difference upon controlling temperature is limited^19^. In addition, perovskite SCs grown at low temperatures present much lower trap density than those at high temperatures^37^. Therefore, high-quality perovskite SCs grown at a constant, low temperature are highly desired.

In this work, we develop a novel room-temperature (RT) liquid-diffused separation induced crystallization (LDSC) method to grow high-quality 3-dimentional (3D) CH_3_NH_3_PbX_3_ (MAPbX_3_, X = Cl, Br, I) and 2-dimentional (2D) (C_4_H_9_NH_3_)_2_PbBr_4_ (BA_2_PbBr_4_), (C_3_H_7_NH_3_)_2_PbBr_4_ (PA_2_PbBr_4_), and (C_6_H_5_CH_2_NH_3_)_2_PbBr_4_ (PMA_2_PbBr_4_) perovskite SCs. The crystallization process initializes from an unsaturated perovskite solution by changing the solution concentration, that is, the solvent escapes out of the solution through spontaneously diffusing into silicone oil. When it reaches saturated, we keep the concentration of the perovskite precursor as a constant and only change the volume in the following process. We select silicone oil as the separation medium as its density is slightly higher than that of solvent but lower than that of perovskite precursor. The solvent diffuses into the silicone oil and escapes out, leading to the formation of oversaturated perovskite precursor solutions. In contrast to controlling the heating or cooling rate, maintaining the precursor solution at RT is apparently much easier. With this knowledge, we establish this facile crystallization route for growing SCs across a wide range of perovskite semiconductors and uncover how to enable to grow high-quality perovskite SCs at RT. We further elucidate the growth kinetics mechanism via the LDSC method in detail. The thickness of the bulk 3D SCs can be tuned from microns to millimeters. Compared with the high-temperature MAPbBr_3_ (HT-MAPbBr_3_) ones, the LDSC-MAPbBr_3_ SC detectors show a lower dark current, faster response speed, higher photocurrent, and X-ray sensitivity. The versatility of our approach provides a generic strategy to grow high-quality perovskite SCs with the selection of suitable solvents and medium materials, which further pushes towards next-generation perovskite optoelectronic applications.

## Results

### Growth kinetics of perovskite SCs

It is noted that the growth temperature and precursor concentration play key roles in determining both the quality and the yield of the resultant SCs^25^. Since solubility is measured in terms of the maximum amount of solute dissolved in a solvent at equilibrium, a solution system can reach oversaturated by varying temperature and concentration. The driving force of crystallization from a metastable perovskite precursor solution is oversaturation, which is determined by its temperature and concentration^41^. Typically, the saturation concentration is essentially dependent on temperature^37^. The saturated concentration of perovskite precursor solutions decreases upon increasing temperature, that is, inverse solubility^27,29^. This has been frequently taken for growing perovskite SCs, which is well known as the ITC method. In our case, however, the volume of the precursor solutions shrinks while the concentration of the perovskite precursors keeps as a constant. The absence of temperature fluctuation leads to better SC quality as demonstrated below.

**Fig. 1:**
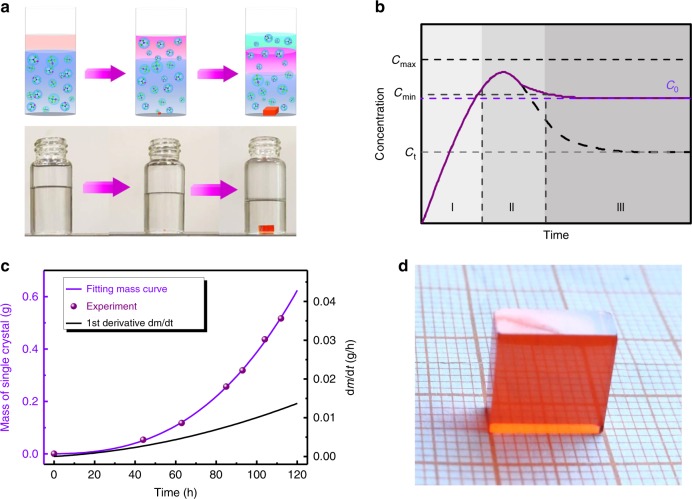
Perovskite single-crystal growth. **a** Schematic illustration of growth diagram of perovskite SCs. **b** Schematic representation of the concentration of solution before and after nucleation as a function of time. *C*_t_ is the solubility, *C*_0_ is the concentration of perovskite solution (*C*_0_ > *C*_t_), *C*_min_ is the minimum concentration for nucleation, i.e., the minimum oversaturation level for nucleation, and *C*_max_ is the maximum concentration for nucleation. The regions I, II, and III represent prenucleation, nucleation, and growth stage, respectively^48^. **c** The mass and mass derivative as a function of the growth time for the MAPbBr_3_ SC in DMF with silicone oil as a medium. **d** Photograph of MAPbBr_3_ SC grown using the RT LDSC method.

Figure 1a schematically illustrates the growth diagram of perovskite SCs at RT. The solvent of the precursor solution spontaneously diffuses into silicone oil. In the meanwhile, the precursor solution evolves to be more concentrated. Then perovskite SCs appear in the oversaturated solution. The thickness of crystals can be tailored by tuning the height of the precursor solution from microns to millimeters (Supplementary Fig. 1). To obtain high-quality SCs, the crystal growth should be maximized and extra nucleation should be suppressed. Besides the MAPbBr_3_ one (shown in Fig. 1d), we obtained MAPbCl_3_, MAPbI_3_ 3D perovskite SCs, and BA_2_PbBr_4_, PA_2_PbBr_4_ and PMA_2_PbBr_4_ 2D layered perovskite SCs using the LDSC method, as shown in Supplementary Fig. 2. The X-ray 2*θ* scan on the facet of the crystal presents diffraction peaks, suggesting a well-orientated MAPbCl_3_, MAPbI_3_, BA_2_PbBr_4_, PA_2_PbBr_4_, and PMA_2_PbBr_4_ crystalline, as shown in Supplementary Fig. 3, which are in line with literatures^28,42–44^.

In a metastable solution system, the crystal growth rate is determined by the deposition rate and diffusion rate. The deposition rate is mainly controlled by the concentration and temperature of the solution, while the diffusion rate is by the concentration gradient of the solution. The crystal seed growth is determined by the deposition rate. To prepare high-quality SCs, the deposition rate should be less than the diffusion rate. When crystal is grown in oversaturation solution, the model can be expressed as^25,45^

1 $$- V\frac{{\mathrm{d}} {\mathrm{\Delta}} {\mathit{C}}} {{{\mathrm{d}}t}}\,=\,V\frac{{\mathrm{d}} {\mathit{M}}}{{\mathrm{d}}t},$$

where *V* is the solution volume, *C* the constant concentration of the saturated solution, and *M* the molar weight of crystal, respectively.

In our case, the temperature and concentration of precursor solutions are kept as a constant. Thus, varying the volume of the solution would tune the concentration gradient. Given that the concentration of the saturated solution is constant, the volume of the solution shrinks as N, N-dimethylformamide (DMF) continues to be extracted. Therefore, the crystal growth rate as a function of the solution volume can be expressed as:

2 $$\frac{{\mathrm{d}}m}{{\mathrm{d}}t} = - \frac{{\mathrm{d}}}{{\mathrm{d}}t}\left[\left( {\left( {V(t) - V(0)} \right) \cdot C \cdot M_{{\mathrm{MAPbBr}}_3}} \right) - m\right],$$

where *m* is the mass of the crystal, *V* the solution volume, *C* the constant concentration of the saturated solution, and$M_{{\mathrm{MAPbBr}}_3}$ the molar weight of MAPbBr_3_, respectively. This LaMer model had been reported to present the formation process of the monodispersed solution^46,47^. In Fig. 1b, the processes of nucleation and growth through the LaMer diagram comprise three stages. At the first stage of prenucleation, DMF diffuses into silicone oil and escapes out, while the monomeric perovskite species initialize to accumulate in solution. Thus, no appreciable nucleation occurs, even when the concentration exceeds the solubility level (*C*_t_) of the perovskite precursor. At the second stage of nucleation, the concentration reaches a critical minimum level (*C*_min_) for nucleation and subsequently the nucleation commences. At the third stage of maximum oversaturation, the nucleation dramatically accelerates as the concentration reaches the maximum level (*C*_max_). The accumulation and consumption of the perovskite precursor are balanced at the crystal nucleation and growth stages. The crystal growth terminates when the concentration of monomers is close to *C*_t_ (Fig. 1b, black dash line)^48^. Note that DMF still keeps diffusing at this stage, in spite of neither accumulation nor consumption of the perovskite precursors. Thus, the precursor concentration is constant once the nucleation occurs (Fig. 1b purple solid line). *C*_0_ is always larger than *C*_t_ when the perovskite solution is oversaturated. The variation of *C*_0_ during crystal growth is negligible. Equation (2) can thus be simplified as

3 $$\frac{\mathrm{d}}{{\mathrm{d}}t} = - \frac{1}{2}C \cdot M_{{\mathrm{MAPbBr}}_3} \cdot \frac{{\mathrm{d}}V(t)}{{\mathrm{d}}t},$$

To figure out the relationship of mass of crystal versus growth time, the mass of crystal and growth time were recorded. In Fig. 1c, the curve of crystal growth was obtained by fitting the experimental data. As a result, the mass derivative with respect to the growth time was calculated. It is worth noted that the rate of crystal growth significantly increases upon the growth time.

We next move to model the seed crystal growth. The crystal growth model is determined by two steps: mass transfer and surface integration. In this model, the solute is transferred from the solution to the crystal interface by the mass transfer process, followed by the solute being integrated into the crystal lattice in the surface integration step. Each step can be described as^45,49,50^

4 $$- \frac{{\mathrm{d}}m}{{\mathrm{d}}t} = r_{\rm{c}} \cdot A_{\rm{s}} \cdot (C - C_{\rm{i}}),$$

5 $$- \frac{{\mathrm{d}}m}{{\mathrm{d}}t} = r_{\rm{i}} \cdot A_{\rm{s}} \cdot (C_{\rm{i}} - C_{\rm{t}})^k,$$

where *r*_c_ is the mass transfer coefficient, *A*_s_ the surface area of SC, *C* and *C*_i_ the solute concentration in solution and at the crystal interface, respectively, *r*_i_ the surface integration coefficient, *C*_t_ the solubility of solute, and *k* the surface integration order. Combining Eqs. (4) and (5), the two-step growth model for a SC can be rearranged as (Δ*C* = *C* − *C*_t_):

6 $$\Delta C = \left( { - \frac{{{\mathrm{d}}m}}{{{\mathrm{d}}t}}\frac{1}{{r_{\rm{i}}S}}} \right)^{\frac{1}{k}} - \frac{{{\mathrm{d}}m}}{{{\mathrm{d}}t}}\frac{1}{{r_{\rm{c}}S}},$$

when Δ*C* = 0 (the concentration is unchanged), the growth rate can be expressed as

7 $$\frac{{{\mathrm{d}}m}}{{{\mathrm{d}}t}} = \left( { - \frac{{\left( {r_{\rm{c}}} \right)^k}}{{r_{\rm{i}}}}} \right)^{\frac{1}{{k - 1}}} \cdot \ S = K \cdot S,$$

where *K* is a constant as the temperature is always at RT. Therefore, the growth rate of SCs is proportional to their surface area. In Fig. 1c, it is obvious that dm/dt sharply increases upon the growth time. In this case, we know that the surface area of crystal is as a quadratic function of time. Combining with the concentration function, the theoretical and measured crystal yield with respect to the growth concentration are calculated and illustrated in Supplementary Fig. 4. We find that the yield at high concentrations is larger than that at low concentrations. The yield reaches up to 92% at 0.5 mol L^−1^, which is the highest reported value so far. Figure 1d displays a MAPbBr_3_ SC obtained using the RT LDSC method. The thickness of the crystals can be tailored from microns to millimeter (38 µm to 4 mm) as shown in Supplementary Fig. 1a–g.

### Characterization of MAPbBr_3_ SCs

**Fig. 2:**
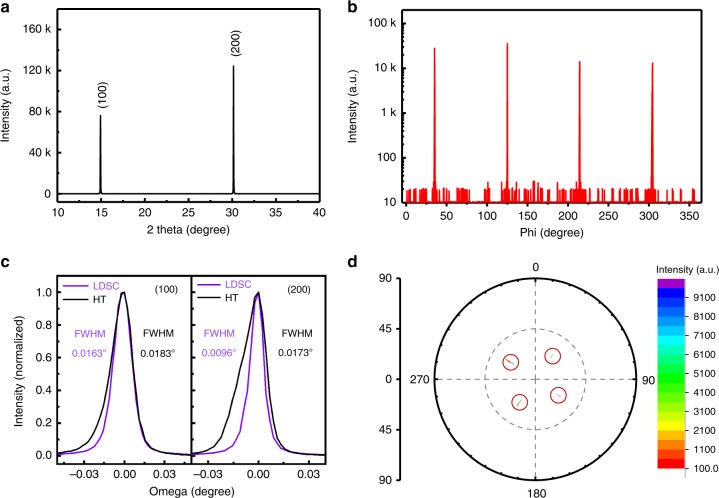
X-ray diffraction of MAPbBr_3_ single crystals. **a** XRD 2*θ* scans of the MAPbBr_3_ SC grown using the LDSC method. **b** Phi scan curve of the (201) diffraction peak of the MAPbBr_3_ SC. **c** High-resolution XRD rocking curve of the (100) and (200) diffraction peaks of the MAPbBr_3_ SCs grown using the LDSC and HT methods. **d** The pole figure of the (201) diffraction peak of the MAPbBr_3_ SC.

The crystallographic structure of MAPbBr_3_ SC grown using the RT LDSC method was investigated with X-ray diffraction (XRD). For comparison, MAPbBr_3_ SCs using the HT method^27^ are also grown and investigated. In Fig. 2a, the XRD 2*θ* scan on its maximal facet presents only (100) and (200) diffraction peaks, suggesting a well-orientated MAPbBr_3_ crystalline. The phi scan curve of the (201) diffraction peak shows that the rotation of the (201) diffraction peak gives four peaks with a 90° interval (Fig. 2b). It is further ascertained that the crystal is quadruple symmetric. Then, the (100) and (200) peaks were further analyzed carefully using high-resolution X-ray rocking curve with fixed 2*θ* at 14.96° of (100) and 30.15° of (200), respectively (Fig. 2c). The full width at half maximum (FWHM) of these two peaks are as narrow as 0.0163° and 0.0096° for the (100) and (200) peaks, respectively. These values are much smaller than the HT method prepared crystals, i.e., 0.0183° and 0.0173° for the (100) and (200) peaks, respectively. Furthermore, we also compared our LDSC method with the reported LTGC method^25^. Interestingly, we found the same trend, that is, low temperature can effectively reduce the FWHM of rocking curves compared with high-temperature methods, indicating enhanced crystallinity. In addition, the FWHM of rocking curve at (200) is 0.0096°, which is slightly narrower than the record value (0.013°) reported for MAPbBr_3_ SCs by Liu et al., which may attribute to the further decreased growth temperature of LDSC at RT, compared with the LTGC method at 60 °C^25,51^. In addition, when the 2*θ* was fixed, the sample was azimuthally rotated from 0° to 360° at a series of tilted angles from 0° to 90°. The appearance of discrete spots in the pole figure instead of rings indicates that the MAPbBr_3_ SC is in order both out-of-plane and in-plane. Figure 2d shows a pole figure of MAPbBr_3_ SC with spots (in red rings) azimuthally separated with 90°.

Next, we move to investigate the optical properties of the MAPbBr_3_ SCs. Supplementary Fig. 5a presents the absorption spectra of LDSC-MAPbBr_3_ and HT-MAPbBr_3_ SCs. Both MAPbBr_3_ SCs exhibit a sharp absorption onset at 595 nm. The optical band gap (2.15 eV) was estimated from the photoluminescence (PL) and absorption spectra, which is in line with literatures^38^. The absorption coefficient *α* was calculated by the absorption spectrum and reflectance spectrum (Supplementary Fig. 6). In the long wavelength region, as shown in Supplementary Fig. 5b, the α of LDSC-MAPbBr_3_ SC is smaller than that of HT-MAPbBr_3_ SC, indicating weaker subband gap absorption and lower trap density.

**Fig. 3:**
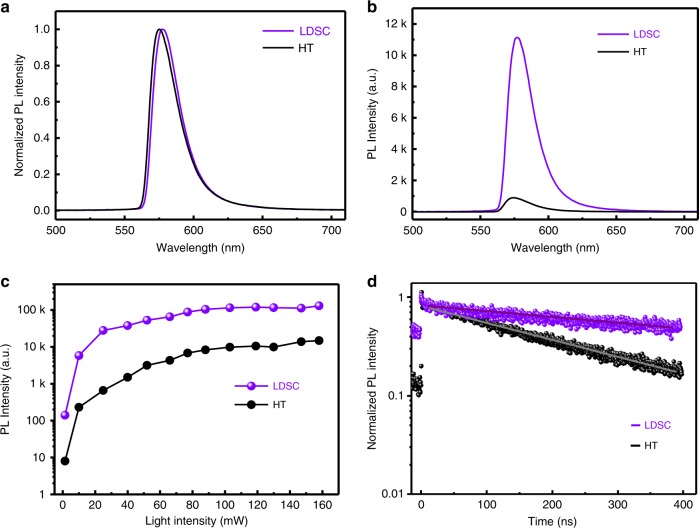
Photoluminescence of MAPbBr_3_ single crystals. **a** Normalized photoluminescence (PL) spectra of LDSC-MAPbBr_3_ and HT-MAPbBr_3_ SC. **b** PL spectra of LDSC-MAPbBr_3_ and HT-MAPbBr_3_ SCs at the same excitation intensity. **c** PL intensity of LDSC-MAPbBr_3_ and HT-MAPbBr_3_ SCs as a function of excitation intensity. **d** Time-resolved photoluminescence of the LDSC-MAPbBr_3_ (violet) and the HT-MAPbBr_3_ (dark) SCs.

The PL peaks of LDSC-MAPbBr_3_ and HT-MAPbBr_3_ SCs locate at 577 nm, with a narrow FWHM of 22 nm (Fig. 3a). However, the PL intensity of both crystals exhibited substantial difference at the same excitation intensity. As shown in Fig. 3b, the PL intensity of LDSC-MAPbBr_3_ is more than ten times higher than HT-MAPbBr_3_, which means that the quality of LDSC-MAPbBr_3_ SC is higher than that of HT-MAPbBr_3_ SC. Furthermore, we investigated the PL intensity as a function of excitation intensity for both SCs. As shown in Fig. 3c, the PL intensity increases rapidly upon the increase of light intensity. The PL intensity of LDSC-MAPbBr_3_ SC is promptly increased and becomes saturated, while the HT-MAPbBr_3_ keeps increasing. We further verified it from the PL intensity derivative as a function of excitation intensity, which can be seen in Supplementary Fig. 7. The results indicate that the nonradiative recombination of the LDSC-MAPbBr_3_ SC is much lower than that of HT-MAPbBr_3_ SC. The recombination dynamics of photoexcited carriers in both LDSC-MAPbBr_3_ and HT-MAPbBr_3_ SCs were investigated with time-resolved photoluminescence (TRPL) spectroscopy (Fig. 3d). The mono-exponential lifetimes can be calculated from the mono-exponential fitting to the PL decay. The LDSC-MAPbBr_3_ SC exhibits significantly longer carrier lifetime (997 ns) in contrast to that of the HT-MAPbBr_3_ SC (235 ns), indicating much lower monomolecular recombination rates and fewer defects. These results demonstrate that superior quality is achieved for MAPbBr_3_ SCs using our LDSC strategy.

**Fig. 4:**
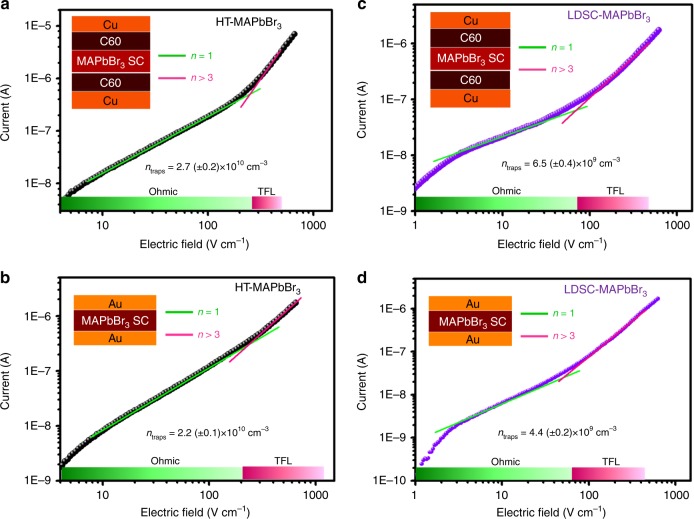
Characterization of single crystals trap density. Characteristic *I–E* curves of **a** electron-only and **b** hole-only devices of HT-MAPbBr_3_, and **c** electron-only and **d** hole-only devices of LDSC-MAPbBr_3_, respectively. A linear ohmic regime (green line) is followed by the trap-filled regime, marked by a steep increase in current (pink line).

We investigated the key metrics of perovskite SCs that directly affect their deployment in optoelectronic applications: carrier lifetime τ, and trap density. The trap density (*n*_traps_) of the MAPbBr_3_ SCs were investigated by the space-charge-limited current (SCLC) method. Typically, the electron-only device structure was (Cu/C60/SC/C60/Cu), while the hole-only one was Au/SC/Au. Figure 4 displays the dark *I–E* characteristics of HT-MAPbBr_3_ SCs (Fig. 4a, b) and LDSC-MAPbBr_3_ SCs (Fig. 4c, d), respectively. Two regions were divided in the experimental data. At low bias voltages, the *I–E* response was ohmic (i.e., linear), as confirmed by the fit to linear dependence (green line). At intermediate bias voltages, a trap-filled region started at *V*_TFL_ where the current exhibited a rapid nonlinear rise and signaled the transition into the trap-filled limit (TFL)—a regime in which all the available trap states were filled by the injected carriers. The trap density (*n*_traps_) was calculated using the following relation^43^:

8 $$n_{\rm{traps}}\,=\,\frac{{2V_{{\mathrm{TFL}}}\varepsilon \varepsilon _0}}{{eL^2}},$$

where *V*_TFL_ is the TFL voltage, *ε* the relative dielectric constant of perovskite (25.5)^52^, *ε*_0_ the vacuum permittivity, *e* the electronic charge, and *L* the thickness of the crystal. The determined electron trap density is 2.7 (±0.2) × 10^10^ cm^−3^ for HT-MAPbBr_3_ SC, while it is only 6.5 (±0.4) × 10^9^ cm^−3^ for LDSC-MAPbBr_3_ SC. The hole trap density is 2.2 (±0.1) × 10^10^ cm^−3^ for HT-MAPbBr_3_ SC, while it is only 4.4 (±0.2) × 10^9^ cm^−^^3^ for LDSC-MAPbBr_3_ SC. Note that the *n*_traps_ of the LDSC-MAPbBr_3_ SC is much lower than that of its polycrystalline counterparts (1 × 10^17^ cm^−^^3^)^25^ as well as those of most state-of-the-art semiconductors, including Si (10^13^–10^14^ cm^−3^)^53^, CIGS (10^13^ cm^−3^)^54^, and CdTe (10^11^–10^13^ cm^−3^)^55^. The ultralow trap density indicates excellent crystal quality for the LDSC-MAPbBr_3_ SC. The essential properties of MAPbBr_3_ SC using various methods at different temperatures are summarized in Table 1. One can conclude that our RT LDSC-MAPbBr_3_ SCs are superior in terms of carrier lifetime and trap density, which are essential for high-performance optoelectronic devices. The ultralow trap density is essential to enable a low dark current, which is critical for achieving high-performance perovskite detectors^17,56^.

**Table 1:** Performance metrics of MAPbBr_3_ SCs with different growth methods.

Method	Temperature (°C)	Trap density (cm^−3^)	Lifetime (ns)	FWHM of rocking curve (°)	Reference
AVC	RT	5.8 × 10^9^	978^a^	–	^19^
LTGC	60	6.7 × 10^9^	815^b^	0.0190	^25^
ITC	80	3.0 × 10^10^	300^a^	–	^38^
CTAC	–	1.39 × 10^11^	–	0.0440	^31^
LDSC	RT	4.4 × 10^9^	997^b^	0.0096	This work

^a^Measurement by transient absorption spectroscopy technique.

^b^Measurement by time-resolved PL spectroscopy technique.

### Characterization of SC-based photodetectors

**Fig. 5:**
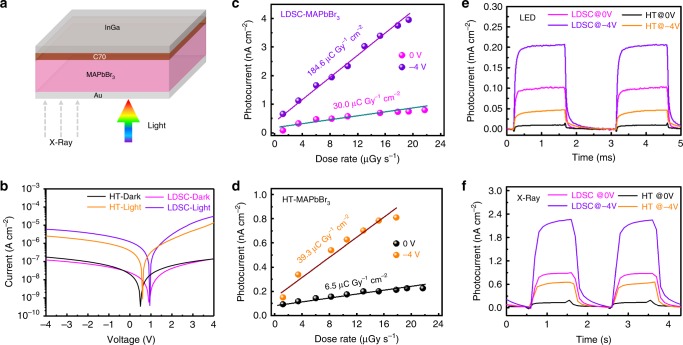
Device performance of MAPbBr_3_ single crystal-based photodetectors. **a** Schematic illustration of the structure of MAPbBr_3_ SC devices. The carriers generated under the illumination of visible light or X-ray. **b** Dark current and photocurrent of MAPbBr_3_ SC devices. X-ray generated photocurrent versus dose rate of LDSC-MAPbBr_3_ SC detector (**c**) and HT-MAPbBr_3_ SC one (**d**), respectively. Current response of MAPbBr_3_ SC device under the illumination of white LED (**e**) and X-ray (**f**) at different biases, respectively.

The electronic properties of perovskite SCs were characterized. The MAPbBr_3_ SC PDs with the structure of InGa/C70/MAPbBr_3_/Au (shown in Fig. 5a) were fabricated. Figure 5a illustrates detector characteristics under the illumination of visible light or X-ray. The MAPbBr_3_ SC PDs exhibit an ultralow dark current. As shown in Fig. 5b, the photocurrent of the LDSC-MAPbBr_3_ SC PDs under the illumination by white LED is as twice as that of the HT-MAPbBr_3_ SC PDs. The photocurrent at negative bias increases drastically for both SC PDs, indicating that a considerable number of carriers are photogenerated. The sensitivity and lowest detectable dose rate are the most important figures of merit to evaluate the performance of an X-ray detector. As shown in Fig. 5c, d the generated photocurrent density signal has a linear relationship with the X-ray dose rates. The X-ray sensitivity was examined by fitting the photocurrent density as a function of the dose rate. For the 2.02-mm-thick LDSC-MAPbBr_3_ SC-based X-ray detector, a sensitivity of 30.0 µC Gy^−1^ cm^−2^ under zero bias is obtained, which is almost as five times as that of 1.42-mm-thick HT-MAPbBr_3_ SC one (6.5 µC Gy^−^^1^ cm^−2^). Furthermore, the LDSC-MAPbBr_3_ SC X-ray detector shows a sensitivity of 184.6 µC Gy^−1^ cm^−2^ at −4 V, much higher than that of HT-MAPbBr_3_ one (39.3 µC Gy^−1^ cm^−2^). In particularly, the detectable X-ray dose rate of LDSC-MAPbBr_3_ SC X-ray detector is lower than 1.2 µGy s^−1^, and achieved a high sensitivity of 184.6 µC Gy^−1^ cm^−2^, which is comparable with reported α-Se X-ray detectors (400 µC Gy^−1^ cm^−2^)^57,58^. However, these α-Se X-ray detectors are operated with high electrical field of 15 V µm^−1^, and our devices require relatively low electrical field of 2 × 10^−3^ V µm^−1^. Figure 5e, f shows on/off photocurrent with respect to time at different biases under the illumination of white LED and X-ray irradiation, respectively. From 0 to −4 V, the photocurrent increase of the LDSC-MAPbBr_3_ X-ray detector is higher than that of the HT-MAPbBr_3_ one. Due to the better charge transport of LDSC-MAPbBr_3_ SCs, the detector at zero bias shows a shorter response time, i.e., the raise and recovery time, of 93 μs for the device than those of HT-MAPbBr_3_ SC one (206 μs) under the illumination of white LED, respectively (Supplementary Fig. 8). Similarly, at −4 V, the LDSC-MAPbBr_3_ SC detector shows a relatively shorter response time of 62 μs compared with HT-MAPbBr_3_ one (205 μs), respectively. We further evaluate the stability of SC detectors through measuring their photoresponse under modulated light illumination without encapsulation in air after 10,000 working cycles. The LDSC-MAPbBr_3_ SC device shows quite stable while the HT-MAPbBr_3_ SC device has a little decrease in photoresponse, as shown in Supplementary Fig. 9. The LDSC-MAPbBr_3_ SC device shows a lower dark current, faster response speed, higher photocurrent and X-ray sensitivity than those of HT-MAPbBr_3_ SC one. These are ascribed to the longer carrier lifetime, and lower trap density of the LDSC-MAPbBr_3_ perovskite, showing superiority optoelectronic applications.

## Discussion

In summary, we report a novel strategy to grow halide perovskite SCs at RT. The perfect combination of solvent selection, silicone oil and growth parameters (e.g., concentration and temperature) allows us to successfully grow various 2D and 3D perovskite SCs using our LDSC method. As the solvent and silicone oil have different density and polarity, they are spontaneously separated from the hybrid precursor solutions. The absence of temperature fluctuation leads to perovskite SCs with ultralow trap density, almost a half of those using the HT method. High-quality MAPbBr_3_ SCs with excellent crystallinity (FWHM of rocking curve <0.0096°), low trap density (4.4 × 10^9^ cm^−3^), and the highest yield (up to 92%) was obtained. As a result, the LDSC-MAPbBr_3_ SC detectors show a lower dark current, faster response speed, higher photocurrent, and X-ray sensitivity. It is expected that the continuous advancement of high-quality perovskite SCs further pushes the development of the new generation of perovskite optoelectronics with superior performance.

## Methods

### Chemicals and reagents

n-Butylammonium bromide (BABr), propylammonium Bromide (PABr), and Benzylammonium bromide (PMABr) were purchased from Xi’an Polymer Light Technology Corp. PbBr_2_ (98%) and silicone oil were purchased from Alfa Aesar. DMF, lead iodide (PbI_2_), dimethyl sulfoxide (DMSO), and methylammonium chloride (MACl) were purchased from Sigma Aldrich. Methylammonium bromide (MABr) and methylammonium iodide (MAI) was purchased from Greatcell Solar Materials Pty Ltd. Acetonitrile (ACN) was purchased from Aladdin. All materials were used as received without further purification.

### Growth of the MAPbX_3_ perovskite SCs

For the LDSC method, MAX (PABr, BABr, and PMABr), and PbX_2_ (X = Br, Cl, I) were dissolved at a 1:1 (2:1) molar ratio in DMF (DMF:DMSO = 1:1, ACN:GBL (gamma-Butyrolactone) = 6:4) solvent. To obtain proper dissolution, the solution was maintained at room temperature under stirring overnight, followed by filtration. After adding an appropriate amount of precursor solution and silicone oil, and placed at RT (MAPbI_3_ for 65 °C). It should be noted that the crystalline takes several days at a constant temperature. A large- and high-quality MAPbX_3_ or BA_2_PbBr_4_, PA_2_PbBr_4_, and PMA_2_PbBr_4_ SCs were obtained, shown in Fig. 1d and Supplementary Fig. 2.

To grow SC at high temperature, MABr and PbBr_2_ were dissolved in DMF solvent with a 1:1 molar ratio. To obtain a proper dissolution, the solution was maintained at room temperature under stirring overnight, followed by filtration. The precursor solution was then transferred into a clean beaker and placed on a hot plate with rising temperature to 90 °C. The HT-MAPbBr_3_ SC was obtained after several hours.

### High-resolution XRD on perovskite SCs

The high-resolution XRD measurements were taken using a Bede D1 with Cu K_α1_ radiation (λ = 1.54056 Å). The film texture was characterized by XRD 2*θ*-scans, *φ*-scans, *omega*-scans, and pole figure measurements with Cu Ka radiation. A pole figure consists of performing a scan about the surface normal (*φ* scan) with fixed 2*θ*, fixed *κ*, and at a range of *φ* scan. One then rotates the sample about the surface normal while measuring the diffracted intensity in steps of *φ* about *κ*_210_. In this way, we can be absolutely sensitive to the in-plane orientation of the crystal structure.

### Absorbance and reflectance spectra measurements

The high-resolution absorption spectra were measured at room temperature with a home-built spectrometer using a monochromator with Xenon lamp as light source. The light intensity was calibrated with certified Si detector, and the photocurrent was recorded with a lock-in amplifier. Reflectance spectra were recorded with an FR-Basic-UV/NIR-HR spectrometer.

### Yield test

First, a series of solution was prepared from 0.05 to 1.5 mol L^−1^. Then, all of the solutions were placed in heated platen until it crystalline completely. The yield curve was obtained by weighing SCs. The yield was calculated by the ratio of the mass of a MAPbBr_3_ SC with the solute of MABr and PbBr_2_.

### Steady-state and time-resolved PL measurements

The TRPL measurements of MAPbBr_3_ SCs were performed using a HORIBA Jobin Yvon IBH Ltd spectrometer. The steady-state PL spectra were recorded with a fiber optic spectrometer (ideaoptics, NOVA-EX) excited with a 445 nm CW laser.

### SCLC measurements

Current as a function of the applied voltage was measured using a Keysight B2912A Precision Sources, using a rather simple geometry with two electrodes on opposite sides of the sample. Ohmic contacts were deposited on opposite sides of the sample by consecutively thermal evaporation of C60/Cu for electron-only and Au for hole-only devices. The sample was kept in a dark environment at room temperature. The device area is 0.04 cm^2^. The thicknesses of HT-MAPbBr_3_ single devices are 2.88 mm for electron-only and 3.02 mm for hole-only, while those of LDSC-MAPbBr_3_ one are 3.12 mm for electron-only and 3.15 mm for hole-only, respectively. The thicknesses of the MAPbBr_3_ SCs were measured by a digital vernier caliper. A nonlinear response was observed and analyzed according to the SCLC theory.

### Device fabrication and measurement

The fabricated device with structure of InGa/C70/MAPbBr_3_ SC/Au. A 35 nm Au electrons and 50 nm C70 layer were thermally evaporated under 2.6 × 10^−6^ Torr. The top InGa electrode was coated on the C70 layer. The current–voltage and current–time characteristics of the PDs were measured by a Semiconductor analyzer B1500A under a white LED excitation and X-ray excitation. The X-ray source is a commercially available SPELLMAN XRB011 tube, with a tungsten anode and 20 W maximum power output. The radiation dose rate was carefully calibrated by using a standard dosimeter (PDM-127B-SH). Fast response was obtained using a white LED modulated with an arbitrary wave function generator (Agilent, 33612A), and the photocurrent responses of these devices were recorded with a digital storage oscilloscope (LeCroy Waverunner 8254). The fast response was obtained using an X-ray source with a mechanical shutter, and photocurrent response of those devices were recorded with a semiconductor analyzer B1500A. The active area is 0.095 cm^2^ of LDSC-MAPbBr_3_ SC device and 0.159 cm^2^ of HT-MAPbBr_3_ SC device. The thickness is 2.02 mm for LDSC-MAPbBr_3_ SC and 1.42 mm for HT-MAPbBr_3_ SC.

## Supplementary information

Supplementary Information

## Data Availability

The data that support the plots within this paper are available from the corresponding author upon request.
