# Supplementary material for: Room-temperature liquid diffused separation induced crystallization for high-quality perovskite single crystals
Source: Nat Commun. 2020 Mar 4;11:1194. doi: 10.1038/s41467-020-15037-x (PMC7055282; doi:10.1038/s41467-020-15037-x)
Supplement: Supplementary file 1 — Supplementary Information [file 41467_2020_15037_MOESM1_ESM.pdf]

Supplementary Information

Room-temperature liquid diffused separation induced crystallization for high-quality perovskite single crystals

Fang Yao, Jiali Peng, Ruiming Li, Wenjing Li, Pengbin Gui, Borui Li, Chang Liu,

Chen Tao, [*] *Qianqian Lin,* [*] *Guojia Fang* [*]

Key Lab of Artificial Micro- and Nano-Structures of Ministry of Education of China,

School of Physics and Technology, Wuhan University, Wuhan 430072, P. R. China.

E-mail: gjfang@whu.edu.cn; q.lin@whu.edu.cn; taochen635@whu.edu.cn

Supplementary Figures

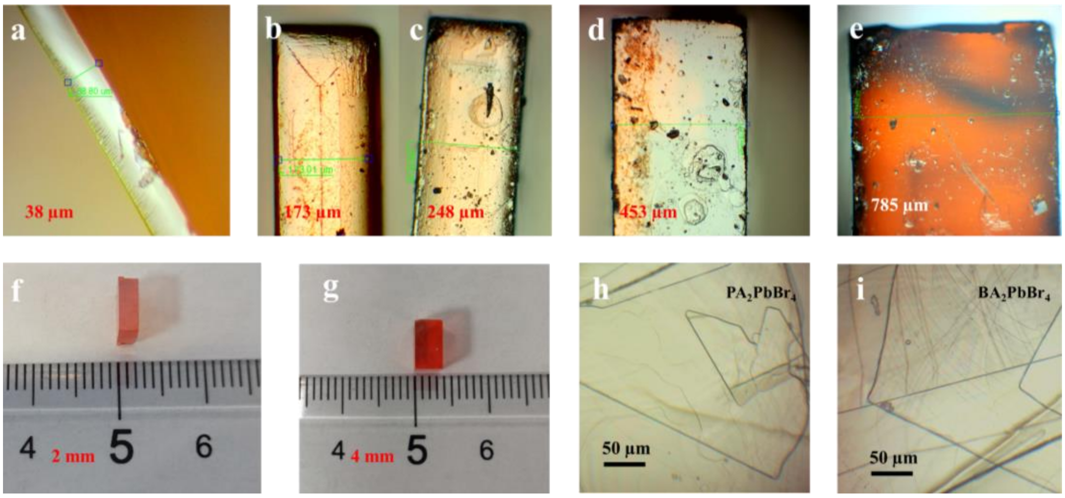

Supplementary Fig. 1 (a) - (g) Microscopy images and optical photographs of different thickness of $MAPbBr_3$ single crystals. Microscopy images of PA_2PbBr_4 (h) and BA_2PbBr_4 (i) single crystals.

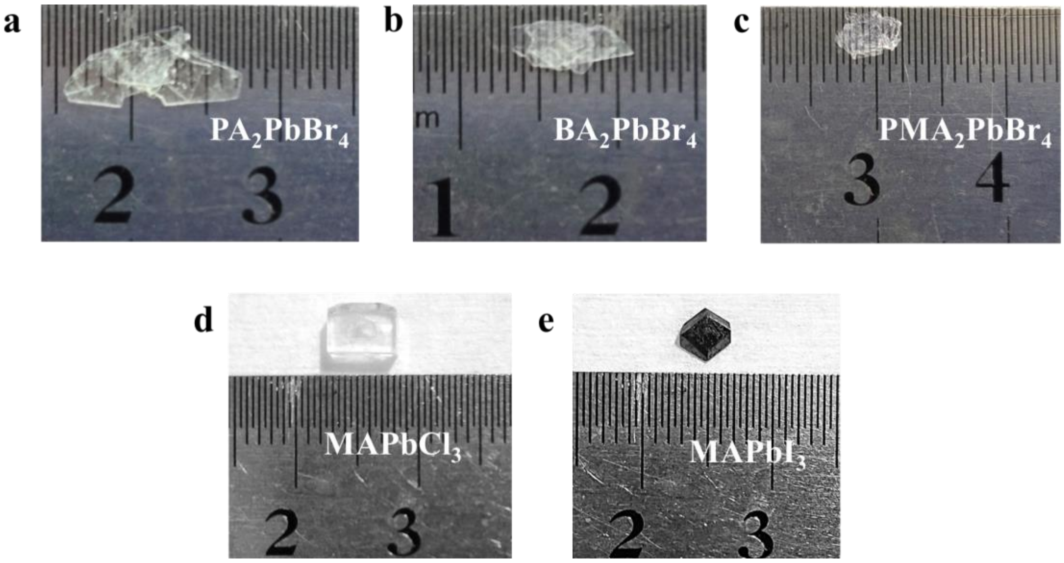

Supplementary Fig. 2 Photographs of perovskite single crystals. (a) BA_2PbBr_4, (b) PA_2PbBr_4, (c) PMA_2PbBr_4, 2D perovskite SCs. (d) $MAPbCl_3$ and (e) $MAPbI_3$ 3D perovskite SCs.

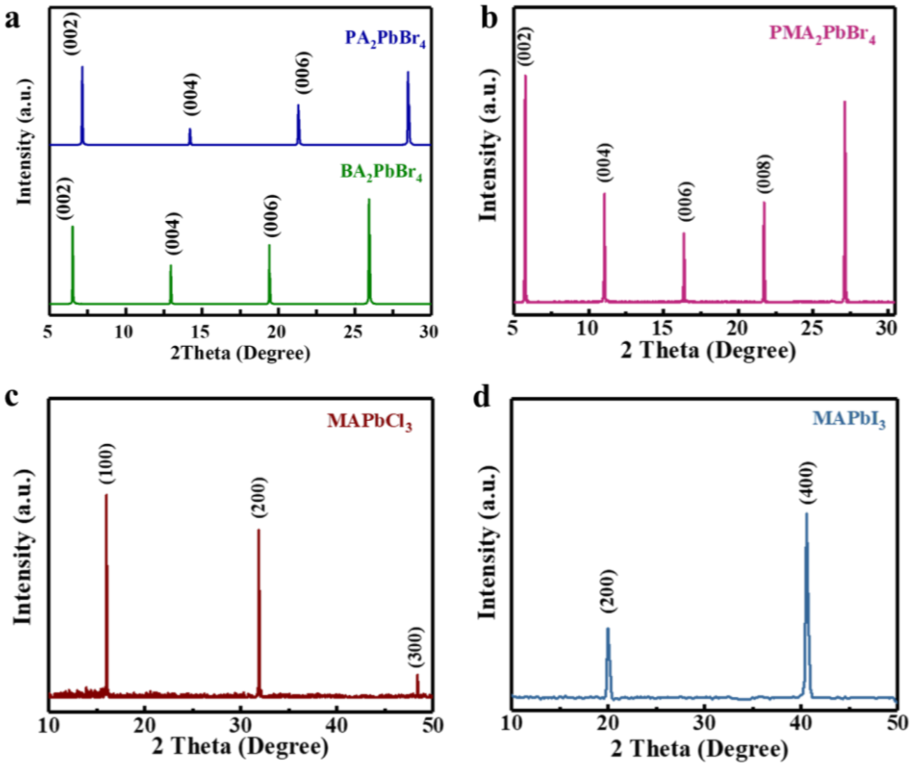

Supplementary Fig. 3 (a) XRD 2 theta scans of the BA_2PbBr_4 and PA_2PbBr_4 single crystals. XRD 2 theta scans of PMA_2PbBr_4 (b), $MAPbCl_3$ (c) and $MAPbI_3$ (d) single crystals. (The XRD data of PMA_2PbBr_4, $MAPbCl_3$ and $MAPbI_3$ single crystals were obtained with Bruker D8)

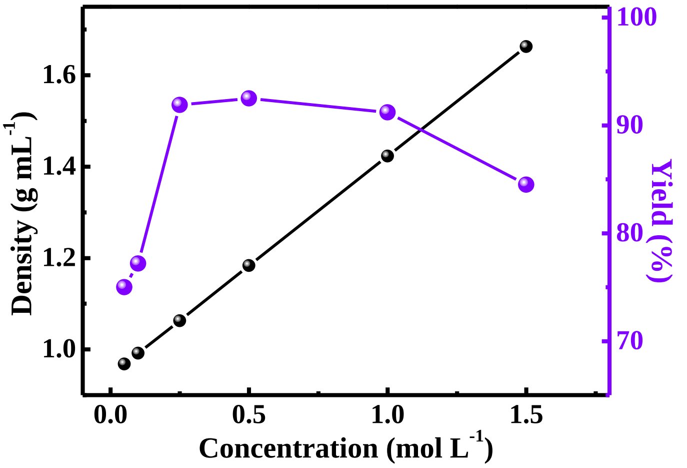

Supplementary Fig. 4 The density and yield with precursor concentration for

$MAPbBr_3$.

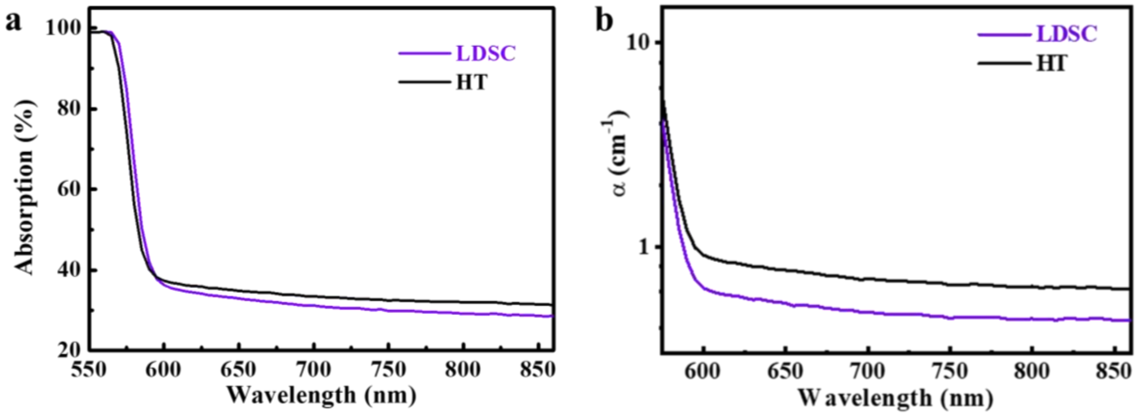

Supplementary Fig. 5 Absorption spectra (a) and absorption coefficient curves (b) of LDSC- $MAPbBr_3$ and HT-$MAPbBr_3$ single crystals.

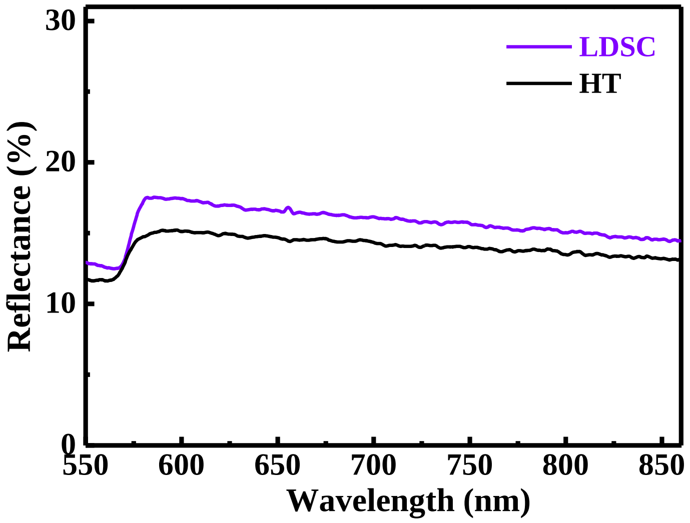

Supplementary Fig. 6 Reflectance spectra of LDSC-$MAPbBr_3$ and HT-$MAPbBr_3$ single crystals.

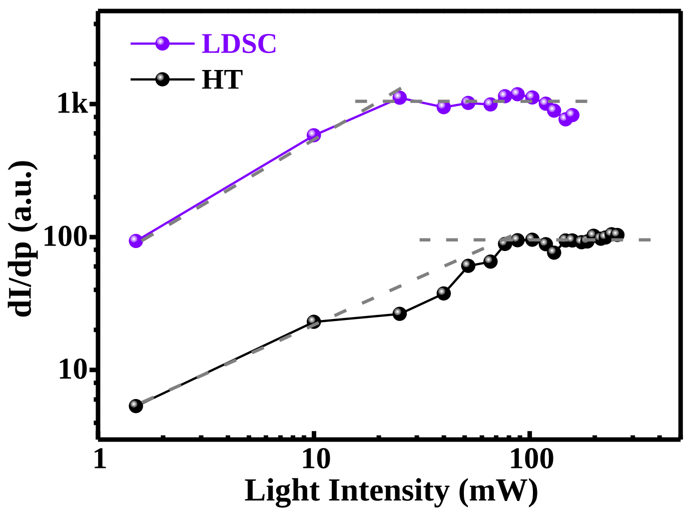

Supplementary Fig. 7 PL intensity derivatives as a function of light intensity for LDSC-$MAPbBr_3$ and HT-$MAPbBr_3$ single crystals, respectively.

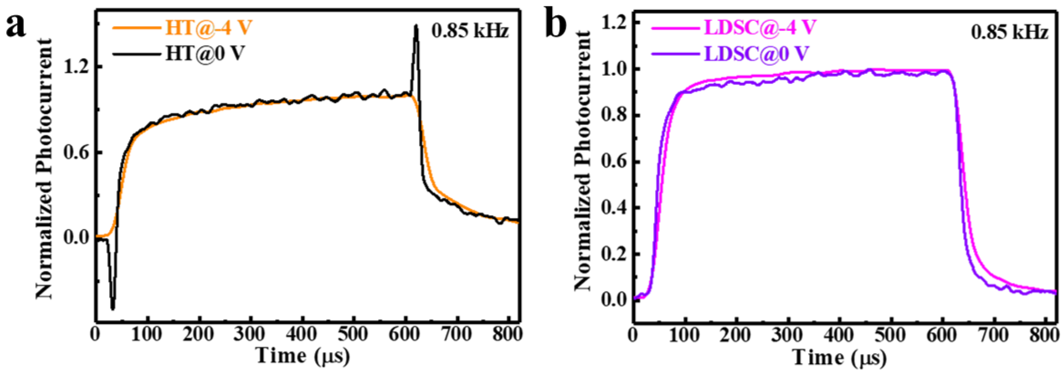

Supplementary Fig. 8 Temporal response of the HT-$MAPbBr_3$ (a) and LDSC-$MAPbBr_3$ (b) single-crystal device under the illumination of white LED at 0 V and -4 V, respectively.

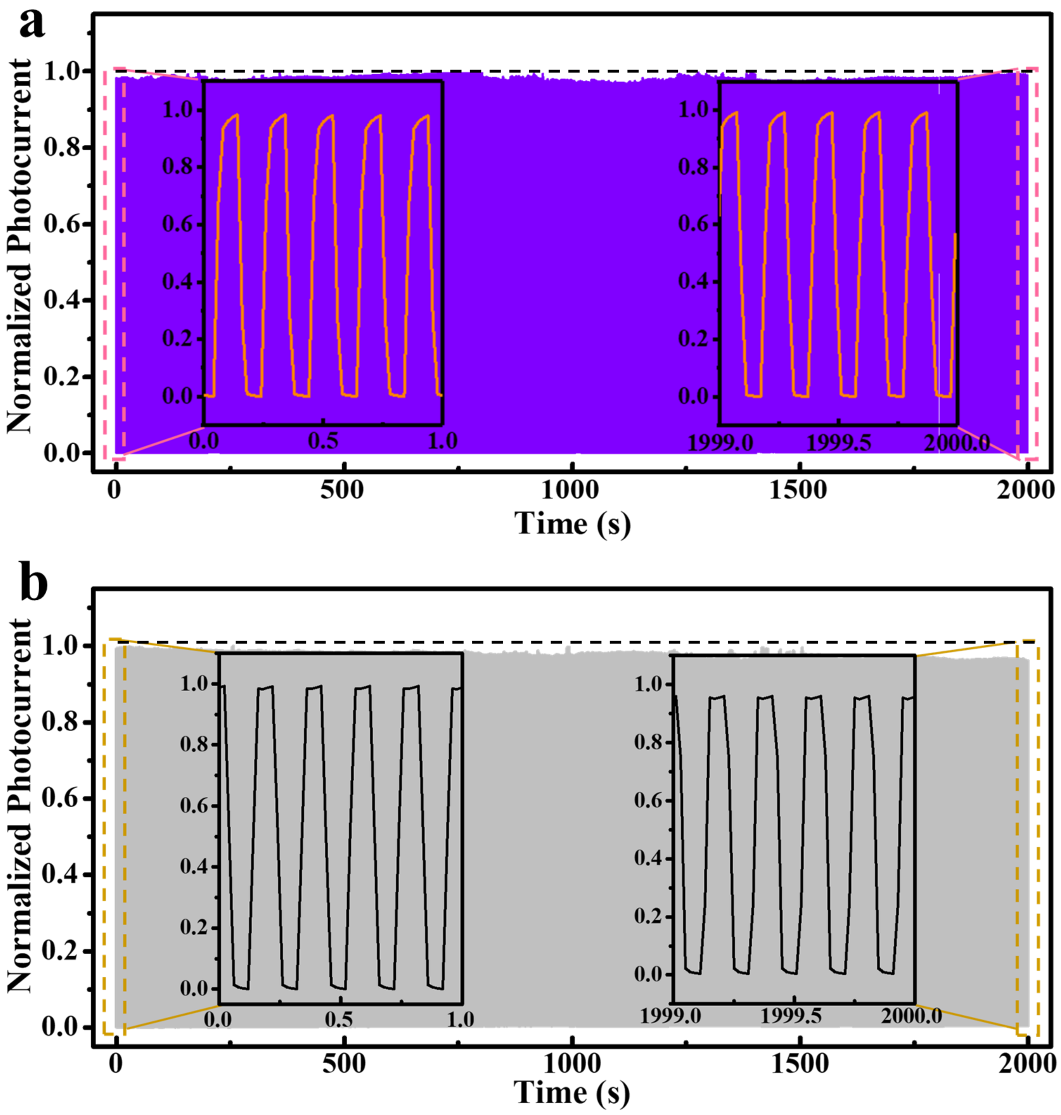

Supplementary Fig. 9 Temporal photoresponse of the LDSC-$MAPbBr_3$ (a) and HT-$MAPbBr_3$ (b) SC device under modulated illumination without encapsulation in air with 10000 working cycles.
